# Awake Craniotomy in Pediatric Neurosurgery: A Case Report on a 12-Year-Old With Recurrent Ganglioglioma

**DOI:** 10.7759/cureus.101278

**Published:** 2026-01-11

**Authors:** Joana Cabral, Diana Sousa, Maria Antunes, Adriano Moreira

**Affiliations:** 1 Anesthesiology, ULS São João (Unidade Local de Saúde São João), Porto, PRT

**Keywords:** craniotomy while awake, drug-resistant epilepsy, intraoperative neurophysiological monitoring (ionm), pediatric anaesthesia, pediatric neurosurgery, dexmedetomidine

## Abstract

Awake craniotomy enables the maximal resection of lesions near the eloquent cortex while preserving neurological function. However, its use in the pediatric population remains limited by challenges of cooperation and anesthetic management. We describe a 12-year-old boy with refractory epilepsy due to the recurrence of a left parietal ganglioglioma previously resected under general anesthesia.

Preoperative assessment included neuropsychological testing and familiarization with the operating room environment. An asleep-awake-asleep technique was used, with propofol and remifentanil for induction, a supraglottic device for airway management, and dexmedetomidine for the awake phase. Language mapping with cortical stimulation identified critical areas and guided tumor resection without complications.

Recovery was uneventful. At one year, the patient remained seizure-free with no cognitive or psychological deficits. This case highlights the feasibility and safety of awake craniotomy in pediatric patients when supported by meticulous preparation and a multidisciplinary team.

## Introduction

Awake craniotomy is a cornerstone in neurosurgical interventions requiring resection of lesions adjacent to critical cortical regions, including those responsible for language or motor function [1-4]. During critical phases of the procedure, the patient is intentionally kept awake to enable real-time assessment of neurological function. This technique facilitates intraoperative functional mapping and guides maximal lesion resection while preserving eloquent cortex [2,4].

Different anesthetic techniques have been described, but monitored anesthesia care and asleep-awake-asleep are the most frequently used. This procedure remains a challenge for anesthesiologists [1-3], as it requires a highly cooperative patient. Appropriate patient selection with neuropsychological assessment, thorough preoperative planning, and effective anxiety management are essential for the success of awake craniotomy [5]. Due to these specificities, its application in children remains scarce and requires close interdisciplinary coordination [6].

Here, we present the case of a 12-year-old boy requiring awake craniotomy for the resection of a recurrent left parietal ganglioglioma, highlighting considerations unique to pediatric patients undergoing this procedure. Through this report, we aim to contribute to the understanding and optimization of awake craniotomy in the pediatric population.

## Case presentation

A 12-year-old boy (61 kg, 172 cm), American Society of Anesthesiologists (ASA) III, presented with drug-resistant focal epilepsy secondary to recurrence of a left parietal ganglioglioma. The lesion had been surgically resected four years earlier under general anesthesia, with histopathological confirmation of a WHO grade I ganglioglioma.

**Figure 1:**
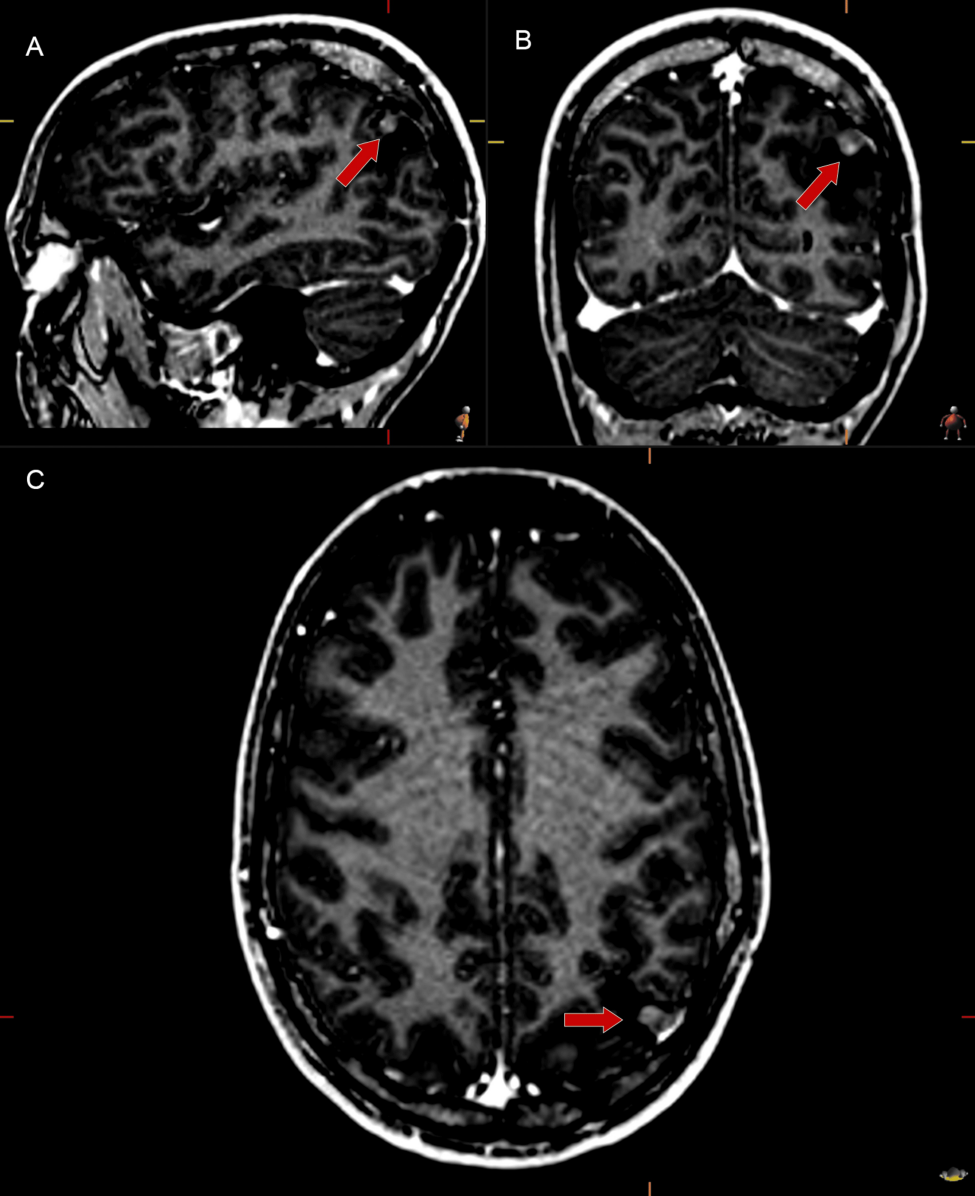
Post-gadolinium contrast-enhanced MRI (sagittal, coronal, axial) showing focal enhancement consistent with recurrent left parietal ganglioglioma (arrows) Post-gadolinium contrast-enhanced brain magnetic resonance imaging (A sagittal, B coronal, and C axial views) demonstrates focal areas of enhancement in the posterior superior aspect of the previous surgical cavity, consistent with recurrent left parietal ganglioglioma (arrows)

Despite an initial period of symptom remission, the patient experienced a recurrence of epileptic seizures two years postoperatively. Brain MRI demonstrated anomalous enhancement foci in the posterior superior aspect of the previous surgical cavity, suggesting tumor recurrence (Figures 1A-1C). Extended video-electroencephalogram monitoring revealed epileptiform activity primarily localized to the left hemisphere, prompting consideration for reoperation.

Given the lesion’s proximity to the eloquent cortex, awake surgery was chosen to allow language mapping and safeguard essential neurocognitive functions. Intraoperative monitoring focused on preserving Wernicke’s sensory speech area.

The patient and his family were thoroughly informed of the surgical and anesthetic plan and potential complications. A preoperative neuropsychological assessment evaluated executive function, attention, memory, language, and motor skills. No baseline deficits were identified. The neuropsychologist was present throughout surgery to support cooperation and guide testing. To optimize comfort, the child was familiarized with the operating room environment and surgical steps in advance.

An asleep-awake-asleep strategy was chosen. General anesthesia was induced with intravenous propofol (Paedfusor model target-controlled infusion (TCI), effect-site targeting) and remifentanil (Minto model TCI, effect-site targeting), titrated according to processed electroencephalography monitoring and clinical response. The airway was secured with a supraglottic device (LMA™ Supreme, Teleflex Medical, Athlone, Ireland). Intraoperative monitoring included standard ASA monitors, invasive arterial pressure, urinary output, and processed electroencephalography (BIS™, Medtronic, Dublin, Ireland). During the asleep phases, bispectral index values were maintained between 40 and 60.

The patient was positioned supine, with the left shoulder elevated and the head rotated 45º to the right. No formal scalp block was performed. Lidocaine 1% with 1:200,000 epinephrine was infiltrated at the Mayfield pin insertion sites and along the incision line, providing adequate analgesia.

Following craniotomy, dura opening, and surgical tumor exposure, continuous electrocorticography monitoring was initiated. Propofol and remifentanil infusions were then discontinued.

A dexmedetomidine infusion (0.3 µg·kg⁻¹·h⁻¹) was initiated. The supraglottic device was removed once the patient was responsive and breathing spontaneously. During the awake phase, the patient remained calm, cooperative, and responsive to language tasks, with preserved spontaneous ventilation and no need for airway support.

Cortical mapping of the language functional area was conducted through direct cortical stimulation at 2-4 mA, successfully identifying critical language areas anterior to the functional supramarginal gyrus. Clinical responses during stimulation, including speech perseveration, semantic paraphasias, and increased drowsiness, indicated functional significance and guided preservation. Neurocognitive tasks (naming, reading, word repetition, counting, verbal fluency) were intermittently administered, ensuring reliable mapping. No seizure activity was recorded. The awake phase lasted approximately three hours and forty-five minutes.

Once surgical resection was concluded, propofol and remifentanil infusions were restarted, dexmedetomidine was maintained, and a supraglottic device was reinserted uneventfully. Closure of the dura mater, bone fixation, and skin closure were performed under general anesthesia. Paracetamol, parecoxib, and morphine were administered intravenously for postoperative analgesia.

No transient or persistent language or cognitive deficits were observed intraoperatively or postoperatively.

The patient was transferred to a pediatric intensive care unit awake, extubated, and breathing spontaneously. His postoperative course was uneventful, with a stable neurological and psychological status. He was discharged on postoperative day seven.

At the one-year follow-up, he remained symptom-free, without psychiatric or neuropsychological issues, reinforcing the safety and feasibility of awake brain surgery in pediatric patients.

## Discussion

Awake craniotomy is the concept of awake brain surgery that allows the matching of motor and language functions to surgically stimulated areas of the cortex [1-3]. The primary goal is to achieve maximal surgical resection of the lesion while preserving brain functions [2,4]. This surgery has different indications such as resection of tumors in the eloquent brain, epilepsy surgery, deep brain stimulation surgery, and interventional pain procedures [2-4].

In the present case, a 12-year-old boy underwent an awake craniotomy for the resection of a recurrent left parietal ganglioglioma causing epilepsy. This decision was driven by the need for precise functional mapping to minimize postoperative neurological deficits and to achieve optimal resection, thereby preventing further recurrence of symptoms. Notably, despite the patient’s young age, a prolonged awake phase was well tolerated, enabling successful language mapping without intraoperative or postoperative complications.

While non-invasive functional mapping methods such as functional MRI have advanced, they may present limitations in pediatric patients, including the potential for overestimating the extent of functional cortex areas [1,7]. Thus, awake craniotomy remains a crucial option, especially when non-invasive methods fail to achieve sufficient mapping. Modern neuroanesthetic techniques and improved perioperative care make this approach feasible [1,2,6,8].

The success of awake craniotomy hinges on two critical factors: appropriate patient selection criteria and the psychological readiness for a surgical procedure performed while awake. The application of this approach is a demanding field for anesthesiologists across all patient types, particularly children, who may experience anxiety, agitation, and non-cooperation due to pain. A preoperative patient interview is essential, as it fosters trust, alleviates anxiety, and enhances communication between the patient and caregivers, which is vital during the awake phase of surgery [5].

At our institution, a multidisciplinary team comprising neurologists, neurosurgeons, anesthesiologists, neuropsychologists, and neurophysiologists works together to evaluate the patient's eligibility for the procedure. Preoperative neuropsychological assessments are performed to evaluate executive function, attention, language, memory, and motor skills. This comprehensive evaluation helps tailor the surgical and anesthetic approach to each child, ensuring they are adequately prepared for the awake craniotomy.

An understanding of the different stages of the surgical procedure and their unique anesthetic management is required. Various anesthetic techniques have been described, with monitored anesthesia care and the asleep-awake-asleep technique being the most frequently used. However, there is no optimal technique, and the most suitable technique must be personalized for each case [1-3]. We opted for the asleep-awake-asleep approach based on its advantages in reducing intraoperative anxiety and agitation, providing adequate analgesia, and controlling brain swelling through mechanical ventilation [1].

In the initial asleep phase, a target-controlled infusion of propofol and remifentanil was administered, enabling neuromonitoring, precise titration, and rapid awakening. Supraglottic devices are well-suited due to their ease of insertion and removal, tolerance in lighter anesthesia, and ability to facilitate mechanical ventilation, thereby ensuring optimal surgical conditions [1].

During the awake phase, a key component of our anesthetic strategy was dexmedetomidine, a highly selective α-2 agonist. Dexmedetomidine was administered at doses of 0.2 - 0.3 µg·kg⁻¹·h⁻¹, providing sedation, analgesia, and anxiolysis without significant respiratory depression [9]. This effectively supported patient comfort and cooperation during the awake phase, without compromising functional language mapping and electrocorticography recordings, as observed in similar cases [9]. Reports describing the successful use of an asleep-awake-asleep approach with dexmedetomidine in pediatric patients are limited, underscoring the clinical relevance of this report.

Despite complications described in the literature, such as seizures, acute brain swelling, respiratory complications, venous air embolism, hemodynamic disturbances, pain, agitation, and failure of the awake procedure [6,8], our surgical procedure was uneventful.

## Conclusions

Awake craniotomy can be successfully performed in carefully selected pediatric patients when supported by meticulous preparation, effective communication, and multidisciplinary collaboration. Comprehensive neuropsychological assessment and preoperative familiarization are essential to ensure cooperation and minimize anxiety during surgery.

The use of an asleep-awake-asleep technique with dexmedetomidine provides a reliable, safe, and comfortable anesthetic strategy that facilitates functional mapping and optimizes surgical outcomes. This case reinforces the feasibility of awake brain surgery in children and its value in balancing maximal tumor resection with preservation of neurological function.
